# Automated identification of Myxobacterial genera using Convolutional Neural Network

**DOI:** 10.1038/s41598-019-54341-5

**Published:** 2019-12-03

**Authors:** Hedieh Sajedi, Fatemeh Mohammadipanah, Ali Pashaei

**Affiliations:** 1 Department of Computer Science, School of Mathematics, Statistics and Computer Science, College of Science, University of Tehran, 14155-6455 Tehran, Iran; 2 Department of Microbial Biotechnology, School of Biology and Center of Excellence in Phylogeny of Living Organisms, College of Science, University of Tehran, 14155-6455 Tehran, Iran; 3 Mathematical and Computer Science Department, Amirkabir University of Technology, Tehran, Iran

**Keywords:** Imaging, Data mining

## Abstract

The *Myxococcales* order consist of eleven families comprising30 genera, and are featured by the formation of the highest level of differential structure aggregations called fruiting bodies. These multicellular structures are essential for their resistance in ecosystems and is used in the primitive identification of these bacteria while their accurate taxonomic position is confirmed by the nucleotide sequence of 16SrRNA gene. Phenotypic classification of these structures is currently performed based on the stereomicroscopic observations that demand personal experience. The detailed phenotypic features of the genera with similar fruiting bodies are not readily distinctive by not particularly experienced researchers. The human examination of the fruiting bodies requires high skill and is error-prone. An image pattern analysis of schematic images of these structures conducted us to the construction of a database, which led to an extractable recognition of the unknown fruiting bodies. In this paper, Convolutional Neural Network (CNN) was considered as a baseline for recognition of fruiting bodies. In addition, to enhance the result the classifier, part of CNN is replaced with other classifiers. By employing the introduced model, all 30 genera of this order could be recognized based on stereomicroscopic images of the fruiting bodies at the genus level that not only does not urge us to amplify and sequence gene but also can be attained without preparation of microscopic slides of the vegetative cells or myxospores. The accuracy of 77.24% in recognition of genera and accuracy of 88.92% in recognition of suborders illustrate the applicability property of the proposed machine learning model.

## Introduction

Myxobacteria are nonpathogenic, free-living bacteria that mainly thrive in terrestrial excosystems as well as marine habitats. They are aerobic and mesophilic bacteria that mainly exist near the surface of the soil. Myxobacteria are Gram-negative with high genomic GC content bacteria that exhibit outstanding characteristics such as the formation of the multicellular fruiting body, gliding motility, predation of microorganisms and cellulolysis. They are considered as a taxonomically distinct group due to their elaborated life cycle that is uncommon in the prokaryotic domain^1^. They are able to lyse other bacteria and yeast cells by predatory behavior. In this process, hydrolyzing enzymes and secondary metabolite molecules secrete to the medium and hydrolyze the prey to consumable nutrients^2^.

During the intricate life cycle, Myxobacteria can produce a resistant and dormant form of cells called myxospore, which is generated, forms vegetative cells inside the fruiting body structures during their complex life cycle. Fruiting bodies of Myxobacteria vary in colour, shape, and size. The size of the fruiting body ranges from 10 to 1000 μm depending on genus and species^1^. The shape of fruiting bodies emanates as spherical, cylindrical, mounds and either hybride form of two or more morphologies like fruiting body of the *Chondromyces* species^3^. Most of the genera produce distinctive and colored fruiting bodies on the surface of the medium that often can be observed through naked eyes. Predominantly, fruiting bodies comprise sporangioles that enclose myxospores in the form of single or clusters. Identification and classification of Myxobacteria extensively dependent on the morphology detail of fruiting bodies, swarming pattern on solid media and shape of the vegetative cells and myxospores.

The correlation between morphology and phylogeny of Myxobacteria investigated by Sproer *et al*. (1999) has shown that the phenotypic classification can provide a consistent basis for the description of neotype species. Accordingly, the findings suggested the classification at the genus level based on morphology is consistent for most of the Myxobacterial genera.

**Figure 1:**
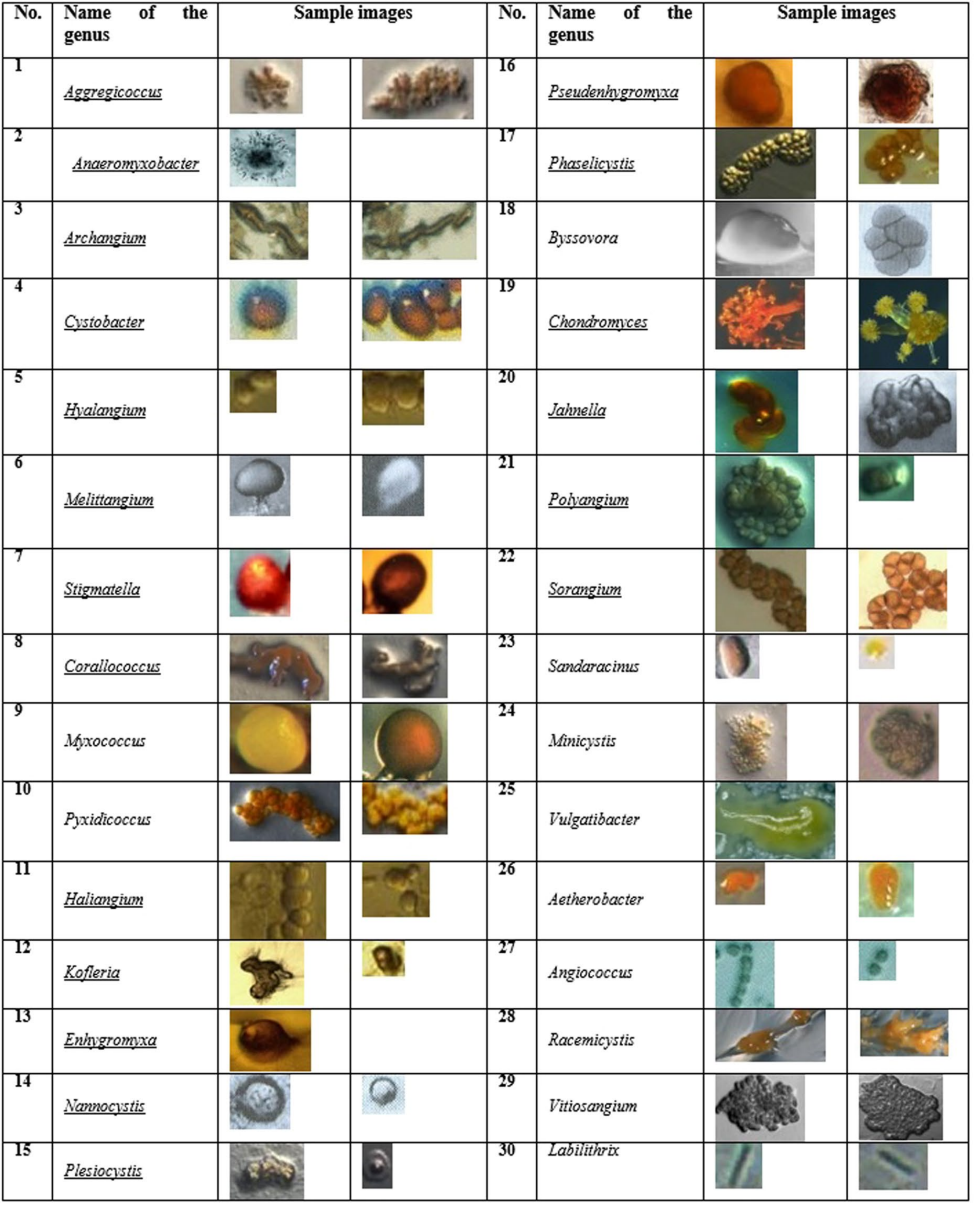
Phylogeny of the order Myxococcales updated on September 2018.

The order *Myxococcales* is placed in δ class of Proteobacteria phylum and consists of three suborders, 11 families, 30 genera and 58 species^4^. The taxonomic classification and phylogeny of the order *Myxococcales* updated on September 2018 is presented in Fig. 1.

There are only few reports on image analysis of Myxobacteria, which have been focused on their swarming motility patterns. Fruiting body formation in Myxobacteria requires swarming by the sensing of signals under starvation conditions. A-signal is involved in the detection of nutrient deficiency in the environment. By entering the cells to a developmental pathway, a large number of cells convert to myxospores and a limited population of cells forms a layer around of myxospores called peripheral cells. On the other hand, C-signal is essential for rippling and differentiation.

It is repoted that 49% of drugs approved between 1981 and 2014 originated from natural products or their semisynthetic derivatives^5^. In the last decade, myxobacteria were recognized to be a valuable source of bioactive secondary metabolites producing novel structure skeletons. Thus, they have received notable attention in drug discovery plans due to the diversity and unique modes of action of their metabolites^6,7^. A large number of myxobacterial metabolites have been reported having antibacterial activity such as myxovalargin^8^, sorangicin^9^, saframycin^10^, sorangiolid^11^, chondrochlorens^12^, and thuggacins^13^ while metabolites like rhizopodin^14^ and chondramides^15^ have shown anticancer activity through interactions with microtubule assembly in the eukaryotic cell lines. In addition, these bacteria represent other rare bioactivities such as anti-malarial, insecticidal, immunosuppressive, and anti-viral activities, etc.^7^.

To screen Myxobacteria for their bioactive metabolites, samples collected from different environments are cultured on several media and differentiated manually. This often makes the whole process of screening labour intensive, time-consuming, and experienced-technicians-oriented^16^. In addition to the 16S rRNA gene sequencing, other genomic approaches such as full genome sequencing, multi-locus sequencing, and metagenomics have been used in their identification^17^ which are time-consuming^18^ and are not needed for the primary identification at the genus level. Therefore, for impressive deduction of labour work during strains screening programs, a high throughput computer-based isolation instead of the experience of an expert is highly appreciated. Artificial pattern recognition or machine learning systems was applied in this study to provide the identification of Myxobacteria at the genus level based on self-experience approach. Consequently, pattern recognition systems implicate algorithms, which their performance will be improved through experience^19^.

No study aimed at characterizing the Myxobacterial genera based on their automated image analysis is reported so far.

In the current study, the development and the application of user-aided image acquisition and automated processing pipeline for the identification of myxobacterial strains based on their descriptive features were achieved. The method proved its robustness toward size and shape variations of the fruiting bodies due to their different maturity state; and changing qualities of images, different technical alterations, and different fields of view. The provided method is fast and accurate, while requires inexpensive assembly of the instrument to assemble the system. In addition, learning of their pattern is active, which results in auto-updating the database. Finally, the application of the method is simple, and there is no need for complex knowledge or years of experience or courses.

Convolutional Neural Network is employed for discrimination between different myxobacterial genera. Furthermore, to increase the accuracy of recognition, the fully connected part of DCNN was swapped with other classifiers. Experimental results illustrate that the proposed model can identify the genera with the accuracy of 77.24% and the higher taxa of suborder with the accuracy of 88.92%.

The structure of the paper is as the following. Section 2 introduces preliminary work includes Extreme Learning Machine (ELM) classifier, On-Line Sequential ELM (OSELM)^20^, Constraint ELM (CELM)^21^, and Convolutional Neural Network. Section 3 describes materials and methods. Experiments and validation are explained in Section 5 and Section 6 concludes the results.

## Preliminary Work

### ELM model

Suppose a set of samples available for building a model is {(*x*_*i*_, *t*_*i*_)|*x*_*i*_ ∈ *R*^*D*^, *t*_*i*_ ∈ *R*^*M*^, *i* = 1, 2, … *N*}. Also, suppose *l* as the number of hidden nodes and *g*(*x*) as the activation function in a multi-layer perceptron neural network. With these assumptions, input weights *W* and the hidden biases *b* can be specified randomly. In this regard, the hidden layer output of ELM can be obtained by Eq. (1):

1 $$H=[\begin{array}{ccc}g({w}_{1}^{T}{x}_{1}+{b}_{1}) & \cdots  & g({w}_{L}^{T}{x}_{1}+{b}_{1})\\ \vdots  & \ddots  & \vdots \\ g({w}_{1}^{T}{x}_{N}+{b}_{1}) & \cdots  & g({w}_{L}^{T}{x}_{N}+{b}_{L})\end{array}]$$

where *w*_*i*_ ∈ *R*^*D*^, *b*_*i*_ ∈ *R*, *i* = 1, 2, …. *L*.

Considering *β* as the output weights, based on the proof presented by Huang *et al*. (Huang *et al*., 2017), the norm of *β* is smaller, and the generalization performance of ELM is more suitable. Consequently, by finding the least square solution of the problem the output weights can be obtained by Eq. (2):

2 $$\begin{array}{rcl}Minimize:{L}_{p} & = & \frac{1}{2}{\Vert \beta \Vert }^{2}+\frac{C}{2}\mathop{\sum }\limits_{i=1}^{N}\,{\Vert {\varepsilon }_{i}\Vert }^{2}\\ subject\,to:h({x}_{i})\beta  & = & {t}_{i}^{T}-{\varepsilon }_{i}^{t},i=1,2,\,\ldots \,N\end{array}$$

where *h*(*x*_*i*_) is the *i* th output vector of the hidden layer, *t*_*i*_ is the *i* th label vector

Based on the Karush–Kuhn–Tucker^22^ theory, Eq. (2) can be expressed by the following Lagrange function:

3 $${L}_{D}=\frac{1}{2}{\Vert \beta \Vert }^{2}+\frac{C}{2}\mathop{\sum }\limits_{i=1}^{N}\,{\Vert {\varepsilon }_{i}\Vert }^{2}-\mathop{\sum }\limits_{i=1}^{N}\,\mathop{\sum }\limits_{i=1}^{M}\,{\alpha }_{i,j}(h({x}_{i}){\beta }_{j}-{t}_{i,j}+{\varepsilon }_{i,j}$$

where each *α*_*i*_ Lagrange multiplier relates to an instance *x*_*i*_. The following set of equations can be calculated by the partial derivative of Eq. (3):

4 $$\begin{array}{c}\frac{{{\rm{\sigma }}L}_{{\rm{D}}}}{{{\rm{\sigma }}{\rm{\beta }}}_{{\rm{j}}}}=0\to {{\rm{\beta }}}_{{\rm{j}}}=\mathop{\sum }\limits_{{\rm{i}}={\rm{1}}}^{{\rm{N}}}\,{{\rm{a}}}_{{\rm{i}}}{\rm{h}}{({{\rm{x}}}_{{\rm{i}}})}^{{\rm{T}}}={{\rm{H}}}^{{\rm{T}}}{\rm{\alpha }}\end{array}$$

5 $$\begin{array}{rcl}\frac{{{\rm{\sigma }}{\rm{L}}}_{{\rm{D}}}}{{{\rm{\sigma }}{\rm{\varepsilon }}}_{{\rm{i}}}} & = & 0\to {{\rm{\alpha }}}_{{\rm{i}}}={{\rm{C}}{\rm{\varepsilon }}}_{{\rm{i}}}\\ i & = & 1\ldots N\end{array}$$

6 $$\begin{array}{rcl}\frac{\sigma {L}_{D}}{\sigma {\alpha }_{i}} & = & 0\to h({x}_{i})\beta -{t}_{i}^{T}+{\varepsilon }_{i}^{T}=0\\ i & = & 1\ldots N\end{array}$$

where *α* = [*α*_1_, …, *α*_*N*_]^*T*^ and the least square solution of *β* is attained by computing the three equations. The answer is as Eq. (7):

7 $$\hat{\beta }={H}^{T}{(\frac{I}{C}+H{H}^{T})}^{-1}T$$

Accordingly, the output function of ELM is as Eq. (8):

8 $$f(x)=h(x){H}^{T}{(\frac{I}{C}+H{H}^{T})}^{-1}T$$

The calculation of dot product in ELM can be replaced by introducing the kernel function *k*(*x*_*i*_ . *x*_*j*_) as Eq. (9). To decrease the computational complexity of high dimensional dot product, it is essential to make sure that *k*(*x*_*i*_ . *x*_*j*_) is simply a mapping method of the relative location of two input examples^23^.

9 $$k({x}_{i}\,.\,{x}_{j})=k({x}_{i}-{x}_{j})$$

### OSELM

In the first phase of OSELM which is boosting phase the Single Layer Feed forward Network (SLFNs) is trained using the primitive ELM method with some batch of training data in the initialization stage and these boosting training data will be discarded as soon as this phase is completed. The required batch of training data is very small, which can be equal to the number of hidden neurons (e.g. for 10 neurons, 10 training samples may be needed to boost the learning).

In the second phase, the OSELM learns the train data one-by-one or chunk-by-chunk and all the training data will be discarded after the learning process on these data is finished^22^.

### CELM

The algorithm of CELM was proposed for constraining the weight vectors {*W*_*j*_|*j* = 1 .…. *L*} from the input layer to the hidden layer by drawing from the closed set of difference vectors of between-class instances, which are the set of vectors correlating the instances of one class with instances of a different class^24^. The pseudo code of CELM training process is illustrated in Algorithm 1. It can be seen that except that the CELM constrains the input connection weights of the hidden neuron, the CELM is similar to the ELM. Experiment results are shown that CELM has a performance with higher efficiency compared to ELM^24^.

**Algorithm 1:**
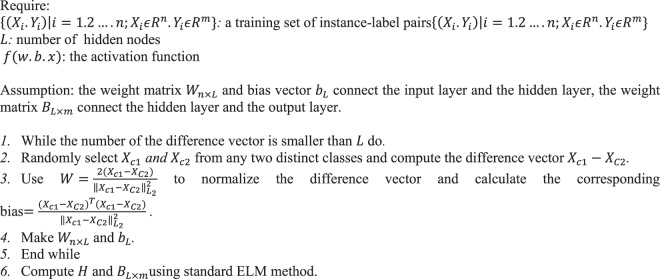
Training process of CELM.

### Convolutional neural network

On a general overview, after feeding images to convolution neural network, which includes several layers of types convolutional, nonlinear, and pooling, the transformed images are delivered to the output layer that can predict a class in classification problems or a single number in regression problems. Typically, the convolutional neural network includes the following layers:

#### Input layer

Usually values for raw pixels of the input image are incorporated into the input layer.

#### Convolution layer

The convolutional layer is the main layer of a CNN. Neurons in this layer are connected to the regions of the image or the previous layer. These areas called the filters to move vertically and horizontally and extract features from the image of the previous layer. For each part, a dot product of the weights and the input is computed, and a bias value is added to it. The step size of filter shift is named *stride*. The number of weights applied for a filter is obtained by Eq. (10) ^25^:

10 $$h\times w\times c$$

where *h* and *w* is the height and the width of the filter, and *c* is considered the number of channels in the input. By using Eq. (11), the number of parameters in a convolution layer is calculated.

11 $$((h\times w\times c+1)\times n)$$

where 1 is for the bias and *n* is the number of filters. The output size of the convolutional layer is calculated using Eq. (12):

12 $$\frac{(I-F+2\times P)}{S}+1$$

where *I* is input size of layer, *F* is filter size, *P* is padding dimension and *S* is stride number.

The layer’s parameters consist of a set of learnable filters. Through the forward flow, each filter is convolved with the input data. Convolution is simply the result of the dot product between the elements of the filter and the input. Accordingly, in the training process of the network, the filters are learnt and are activated when it faced with some specific features at some spatial positions in the input.

A neuron in CNN investigates a small region in the input and shares parameters with neurons in the same activation map.

#### Batch normalization layer

This layer normalizes its inputs x_i_ by estimating the mean μ_B_ and variance ${\sigma }_{B}^{2}$ of a mini-batch and on each input channel. Afterward, it normalizes the activations using Eq. (13):

13 $$\widehat{{{\rm{x}}}_{{\rm{i}}}}=\frac{{{\rm{x}}}_{{\rm{i}}}-{\mu }_{{\rm{B}}}}{\sqrt{{{\rm{\sigma }}}_{{\rm{B}}}^{2}}+\epsilon }$$

when the mini-batch variance is very small, ${\epsilon }$ improves numerical stability. To allow for the possibility that inputs with zero mean and unit variance are not optimal for the layer that follows the batch normalization layer, the batch normalization layer further shifts and scales the activations Eq. (14):

14 $$\,{y}_{i}=\gamma \widehat{{x}_{i}}+\beta $$

Here, the offset ***β*** and scale factor ***γ*** are learnable parameters that are updated during network training. At the end of the learning process, batch normalization layer calculates mean and variance over the full training set and stores them in sequence in trained mean and trained variance properties^25^.

#### Pooling layer

Pooling layer does a form of down sampling. There are several non-linear functions to implement pooling among which max-pooling is the most common. It partitions the input image into a set of non-overlapping rectangles and, for each such sub-region, outputs the maximum. The intuition is that the exact location of a feature is less important than its rough location relative to other features. The pooling layer serves to progressively reduce the spatial size of the representation, to reduce the number of parameters and amount of computation in the network, and hence to also control overfitting. It is common to periodically insert a pooling layer between successive convolutional layers in CNN architecture. Pooling layers provide a form of translation invariance. Specially max-pooling across rotated/scaled database images gains rotation/scale invariance. The *Poolsize* property determines the size of the rectangular regions. The output size of a pooling layer with input size *InputSize* is as Eq. (15):

15 $$Output\,Size=\frac{(inputsize-poolsize+2\times paddingsize)}{stride}+1$$

#### Activation layer

This layer applies the non-saturating activation function. It increases the nonlinear properties of the decision function and of the overall network without affecting the receptive fields of the convolution layer. ReLU is the abbreviation of Rectified Linear Units. ReLU is preferable to other functions, because it trains the neural network several times faster without a significant penalty to generalization accuracy^26^. Other functions are also used to increase nonlinearity, for example, the saturating hyperbolic tangent and the sigmoid function.

#### Fully connected layer

The fully connected layer is a traditional Multi-Layer Perceptron that uses a Softmax activation function in the output layer. The term “Fully Connected” implies that every neuron in the previous layer is connected to every neuron on the next layer. The result of this layer is a vector of 1 × 1 × n, where *n* is the number of classes. After several convolutional and pooling layers, the classification in the neural network is done via fully connected layers. This layer(s) is the part that learns supervisory in contrast to the convolutional, pooling and activation layers that learn nonsupervisory.

#### Softmax layer

This layer receives the output of the previous (fully connected) layer and converts it to a probability distribution on the classes. This is done through Eq. (16):

16 $$p({c}_{r}|x.\theta )=\frac{p(x.\theta |{c}_{r})p({c}_{j})}{{\sum }_{j=1}^{k}\,p(x.\theta |{c}_{j}|)p({c}_{j})}=\frac{{\exp }({a}_{r}(x.\theta ))}{{\sum }_{j=1}^{k}\,{\exp }({a}_{r}(x.\theta ))}$$

where 0 < *p*(*C*_*r*_|*x*.*θ*) ≤ 1 and $\mathop{\sum }\limits_{j=1}^{k}p(x.\theta |{c}_{j})p({c}_{j})=1$. Also ${a}_{r}=\,\mathrm{ln}(p(x.\theta |{c}_{r})p({c}_{r}))$, *p*(*x*. *θ*. *c*_*r*_) is the conditional probability of the sample given class *r* and *p*(*c*_*r*_) is the class prior probability^27^.

## Materials and Methods

The details of preparing MYXO.DB and the proposed method are described in this section.

### Classification of Myxobacteria based on the appearance of the fruiting bodies

The appearance of the fruiting body that harbors the myxobacterial spores (myxospores) can be categorized as Table 1. These morphological characteristics are currently used by expert researchers to distinguish the genera from each other. Therefore, this morphological identification key demands the deep understanding and visualization skill of the researchers in order to lead them to the right genus.

**Table 1:** The macro-morphological specification of Myxobacterial genera used in observational identification.

Family	Genus	Sporangiole^¥^	Characteristics of the Fruiting Body				Characteristics of the Swarm		
			Stalk	Shape of the fruiting body	Size (μm)	Texture	Color and shape of the swarm	Swarmedge	Agar corrosion
*Cystobactereaceae*	*Archangium*	−	−	Variable in size and shape, strings of myxospores in hardened slime	50–1000	Hard	Branched radial veins	Flame like	−
	*Cystobacter*	+	−	Rounded, elongate, or coiled singly or in groups	50–180	Hard	Tough slime sheet with veins	Flame like	−
	*Hyalangium*	+	−	Small spherical sporangioles with glassy shape	35–45	Glassy	Yellow or brown/Thin, tough slime sheet with very fine veins	Fine veins	−
	*Melittangium*	+	+	Semispherical sporangiole like a mushroom cap	50–100	Soft	Bright yellow/Slime sheet and radial veins	Flare- to flame-like	−
	*Stigmatella*	+	+	Spherical sporangioles singly or in clusters	300–350	Hard	Yellow/Tough slime sheet with oscillating waves	Flare like	−
	*Vitiosangium*	−	−	Oval to Bean shaped, solitary mounds	20–200	Soft	Coherent swarm with scattered ripples	Flare like	—
*Myxococcaceae*	*Corallococcus*	−	−	Coral, hornlike, often solitary	20–1,000	Hard	Colorless/thin and transparent	Flares, flames	−
	*Myxococcus*	−	+^|^	Rounded to oval, often solitary	50–200	Soft	Colorless to shade of orange and yellow/thin, film-like	Flares, flames	−
	*Pyxidicoccus*	+	−	Ovoid clusters	30–80	Hard	Colorless/thin, film-like	Flares, flames	−
	*Aggregicoccus*	−	−	Spherical fruiting body-like aggregates	ND	Soft	Transparent swarms, wavy, rippling structures	Intricate veins on edges	−
	*Angiococcus*	+	−	Spheroidal sporangioles	30–40	Soft	Thin, spreading swarm of gliding cells	Flare-like	−
*Anaeromyxobacteraceae*	*Anaeromyxobacter*	+	−	Polyhedral or spherical solitary or cluster	ND	Soft	—	—	−
*Vulgatibacteraceae*	*Vulgatibacter*	−	−	Fruiting body-like aggregates	ND	Soft	No swarming	unstructured	−
*Polyangiaceae*	*Byssovora*	+	−	Polyhedral sporangioles in sorus	220–560	Soft	Pseudoplasmodial thin layer	Fanlike	+
	*Chondromyces*	+	+	Sessile, spectacular, complex, and elegant miniature tree- or flowerlike fruiting body	1000	Hard	light orange and burrow/Thin, filmlike, transparent	Fanlike	+
	*Jahnella*	+	−	Coils shape sporangioles in cluster	60–90 × 80–120	Tough	Orange/Scattered long veins	Bands in agar	+
	*Polyangium*	+	−	Oval to polyhedral sessile sporangioles, arranged in a cluster or solitary	50–400	Soft	Pseudoplasmodial swarm	Fan-shaped	+
	*Sorangium*	+	−	Ovoid to polyhedral sporangioles in cluster and chain	20–30	Hard	Yellow or orange/Soft radial veins	Curtain-like	+
	*Aetherobacter*	+	−	Fascicles in chains or rolls in aggregates	50–3000	Soft	Swarm forms ring- or halo-like colonies	Coherent migrating cells	+
	*Minicystis*	+	−	Small fruiting bodies, ovoid sporangioles	4.0–12.0	Soft	Swarm appears film-like, thin and transparent	unstructured, with loose migrating cells sometimes with tiny flares	−
	*Racemicystis*	+	—	Varying size	200–800	Tough	Orange to beige	Sweep like	−
*Sandaracinaceae*	*Sandaracinus*	−	−	Fruiting body-like aggregates sessile and irregular	50–150	Soft	Orange/shallow wave depressions	Cell mounds at the end	−
*Phaselicystidaceae*	*Phaselicystis*	+	−	Bean, sausage, or ovoid shaped sporangioles	20 × 25, 49 × 56	Tough	Tough, slimy net-like veins	Flame- or flare-like	+
*Labilithrichaceae*	*Labilithrix*	−	−	Raised colonies instead of the Fruiting body	ND	Soft	Slimy	Hairy-like	−
*Nanocystacea*	*Enhygromyxa*	−	−	Fruiting body like aggregates (Rounded, hump, globular)	100–150	Soft	Colorless/light orange to red/delicate slimy veins	Flare to pseudoplasmodium	+
	*Nannocystis*	+	−	Spherical, oval to short sausage-shaped sporangiole	6 × 3.5–110 × 0	Hard	Excavated/deep tunnels	Trails or fine wave	+
	*Plesiocystis*	−	−	Fruiting-like body aggregates	100–500	Soft	Thin, transparent, pseudoplasmodium	Flare-like	+
	*Pseudenhygromyxa*	−	−	Fruiting-like body aggregates	50–800	Soft	Colorless to pale peach/slimy veins	Flare-like	+
*Kofleriaceae*	*Kofleria*	−	−	Yellow knobs in agar or on surface	ND	Soft	Yellow/film-like with radial veins	Lateral rim	−
*Haliangiaceae*	*Haliangium*	+	−	Fruiting-like aggregate or sessile oval-shaped sporangioles	15–150	Soft	Colorless to yellow shades/thin, film-like	Flare- to flame-like	+

^¥^Sporangiole: Packages of myxospores.

^+^Indicate the presence.

^−^Indicate the absence.

ND: Not Distinguishable.

### Selection of the stereomicroscope images of the typical fruiting bodies

Morphological features of the spore producing structures called fruiting bodies were selected based on their typical descriptive morphology from the valid publications. The images were selected based on the descriptive literature and the number of investigated genera was 30 as exists in the order of *Myxobacteria* at the time of conducting the experiment.

During the process of pattern recognition, the features that describe the fruiting bodies including shape, size, intensity, and texture were extracted from the typical fruiting bodies of all 30 genera of this order.

### Preparing the dataset of MYXO.DB

Since the source of images were quite different in terms of the parameters including contrast, resolution, size, illumination, having noise and capturing camera aspects, designing an automatic image processing method for automatic segmentation of samples from images was complex. Therefore, to form a dataset for automatic recognition of Myxobacteria, the single samples or compact fruiting bodies were cropped manually. The genera, which produce single fruiting body, were considered individually, while the genera that does not form concrete fruiting bodies are not produced, their swarm pattern was considered.

**Figure 2:**
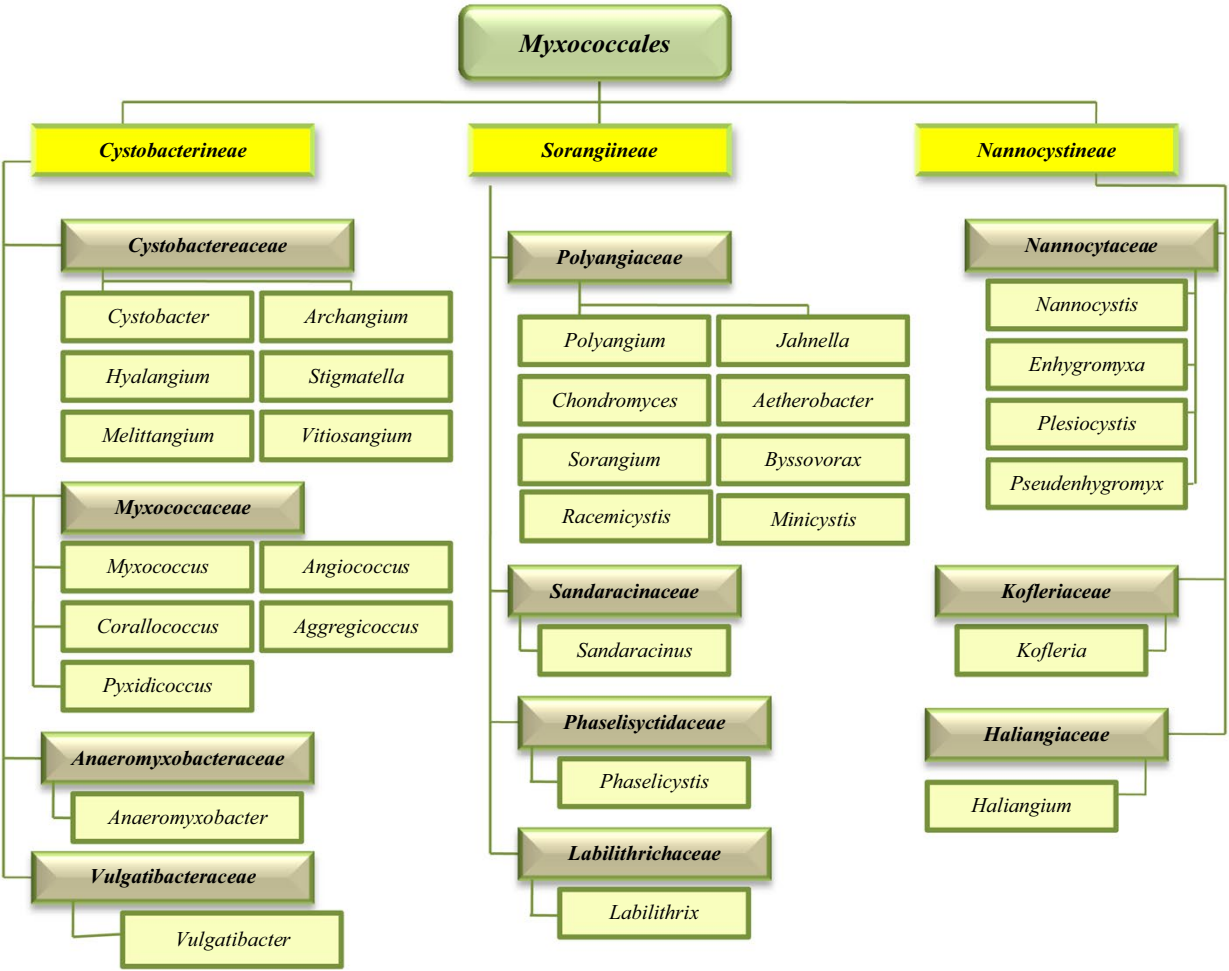
The representative images of each genus in MYXO.DB.

As mentioned before, identification and classification of Myxobacteria dependents principally on the morphology of fruiting bodies, swarming pattern on medium, shape of vegetative cells and myxospores shape. In preparing the dataset of MYXO.DB, morphology of fruiting bodies was analyzed. Furthermore, the colony morphology for some Myxobacteria was considered. Table 2 represents the number of single fruity bodies, which could be extracted from each genus of the 30 classes. Some sample derived images of each genus of MYXO.DB are illustrated in Fig. 2.

**Table 2:** The quantity of each class in MYXO.DB, which contains 322 samples and 30 classes.

No.	Name of the genus	No. of images	No.	Name of the genus	No. of images
1	*Aggregicoccus*	11	16	*Pseudenhygromyxa*	4
2	*Anaeromyxobacter*	1	17	*Phaselicystis*	13
3	*Archangium*	8	18	*Byssovora*	3
4	*Cystobacter*	10	19	*Chondromyces*	17
5	*Hyalangium*	9	20	*Jahnella*	11
6	*Melittangium*	9	21	*Polyangium*	9
7	*Stigmatella*	14	22	*Sorangium*	21
8	*Corallococcus*	15	23	*Sandaracinus*	14
9	*Myxococcus*	38	24	*Minicystis*	20
10	*Pyxidicoccus*	13	25	*Vulgatibacter*	1
11	*Haliangium*	5	26	*Aetherobacter*	14
12	*Kofleria*	10	27	*Angiococcus*	9
13	*Enhygromyxa*	1	28	*Racemicystis*	11
14	*Nannocystis*	14	29	*Vitiosangium*	6
15	*Plesiocystis*	8	30	*Labilithrix*	3

As mentioned in the previous section for learning the difference between distinct types of Myxobacteria and the similarities within the samples of a certain genus, an adequate number of samples should be available. In this regard, some classes with a small number of instances have not been considered in some experiments. In this regard, two datasets with a smaller number of classes were constructed.

MY25 dataset is a subset of MYXO.DB, which contains 313 images, which are categorized into 25 classes. In this dataset, the classes with less than four samples have been removed from the MYXO.DB.

In MY22 dataset, the MYXO.DB dataset is augmented and the number of instances increased by cropping some sub images from each image sample. The augmented dataset contains 629 images, which are categorized into 22 classes. In this dataset, the classes with less than eight samples have been omitted.

To recognize the suborder of each strain, MYCategories dataset is built which contains 319 images samples of MYCategories were categorized into three classes includes *Cystobacterineae, Sorangiineae* and *Nannocystineae*.

By viewing the samples, it can be concluded that some classes are similar to each other for example *Stigmatella* and *Myxococcus* in terms of the morphology of fruiting body. Such similarities can encounter the automatic recognition systems with a notable challenge. On the other hand, a few number of samples for some classes like *Anaeromyxobacter*, *Myxococcus* and *Vulgatibacter* prevents a pattern recognition system from automatic learning. Like learning of a human being child, for an efficient machine learning process, there should be sufficient training samples or experiences^28^.

Further, the color of the fruiting body and the background, and the shape and orientation of colonies are diverse in different samples of a certain class.

### Preprocessing

The discriminating features of Myxobacteria are their shape and texture. Their color can be altered by some culture conditions; therefore, color is not a reliable distinguishing feature for recognizing different types of Myxobacteria.

Some descriptors of the object morphology like area, convex area, roundness etc. denote the size and shape approximately. Consequently, before imposing the sample images to any pattern recognition system, initially, all the images should be converted to gray scale image to drop the color information.

In addition, all the images have been normalized to the size of 100 × 100 pixels because of the below reasons:

- Capturing device for each image of Myxobacteria has a certain property.
- The resolution and the amount of the noise in images are dissimilar.
- Different level of maturity causes diverse size of individuals or colonies.
- The magnification magnitude of the microscope in image capturing may be disparate.

The input of the proposed method is normalized gray scale images. Equation (17) is used for converting a colored image *I* in RGB color space to gray scale image *I*_*g*_ ^29^ where *I*_*R*_, *I*_*G*_ and *I*_*B*_ are the image *I* in Red, Green and Blue color plate and *I*_*g*_ is the resulted gray level image.

17 $${I}_{g}=0.2989\times {I}_{R}+0.5870\times {I}_{G}+0.1140\times {I}_{B}$$

### Feature extraction by convolution neural network

In this paper, for automated recognition of different fruiting bodies a CNN classification model is designed. By the emerging application of deep learning in computer vision applications, extracting the features through the CNNs is beneficial for producing general image descriptors. CNNs resulted in high efficiency in many image classification applications^30^. CNNs were inspired by biological processes in which the connectivity pattern between neurons is deduced by the organization of the animal visual cortex^31^. The major ascendancy of CNNs is partial independence from previous information and human effort in feature design.

A CNN includes a stack of different types of layers that convert the input data into an output value or label. After a brief description of the applied algorithm in this study, the structure of the designed CNN for automatic recognition of Myxobacteria fruiting bodies is described as the following.

#### Input layer

The input of this layer is images with equal size and labeled. In our modeling, raw pixels of images with the size of 28 × 28 are fed into the input layer.

#### Convolutional layer

Our model comprised four convolution layers. In the first convolution layer, 16 filters, in the second layer, 32 filters, in the third layer, 64 filters, and in the last layer 128 filters are used. The size of filters is considered 3 × 3 with the padding size of one.

#### Batch normalization layer

In order to normalize the extracted features using the convolution layer, a batch normalization layer is considered following each convolution layer. The applied model contains four convolutional layers and one batch normalization layer after each one.

#### Rectified linear unit (ReLU) layer

This layer activates max (0, x) activation function on each neuron, that causes the negative values to be converted to zero. The proposed model comprises four ReLU layers.

#### Pooling layer

Four max-pooling layers are considered in the model of this study. The pool size in these layers as well as the stride size of each layer, is [2 2]. Therefore, the size of the output from the first, the second, the third, and the fourth pooling layers are 14, 7, 3, and 1, respectively.

#### Fully connected layer

The proposed model encompassed one fully connected layer.

#### Softmax Layer

The ultimate layer in the model is Softmax layer. This layer is placed after fully connected layer to replicate the outputs of fully connected layers to a probability distribution on the defined classes.

The features extracted from the convolutional and pooling layers comprise descriptors reflecting data on the acquired inside shape of the fruiting bodies (e.g., area of the convolutional mask relative to the colony size, mask area in the center and border of the colony, object sizes, number of objects in the mask, and deviations).

**Figure 3:**
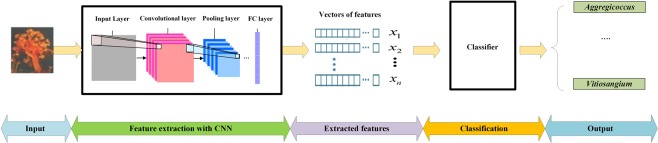
The structure of the proposed CNN for automatic identification of the genera.

The combined feature set obtained from the last layer of unsupervised part of CNN assists as a quantitative signature of the phenotype of the Fruiting bodied. The images from the same genus or belonging to the same phenotypic class share some matching characteristics from the various existing features. The structure of the designed CNN for automatic recognition of the genera is displayed in Fig. 3.

In each convolutional layer, the input convolves with some different filters. In the next pooling layer the size of convolved images reduced to smaller images. This routine is continued to the end of the first phase of CNN.

The first part of a CNN is a feature extraction phase, which also called unsupervised phase. In this phase, the features, which are corresponding to texture and shape, are learned. The output of this phase is diverted to the second part. The second part is a classifier also called supervised phase.

The learning algorithm of the second part is stochastic gradient descent with momentum^32^.

### Extreme convolution neural network (E-CNN)

In this algorithm, in the first step, the features of images are extracted using the convolution neural network and then these features are sent as inputs for Extreme learning machines algorithm.

#### Feature extraction

As we see in the second method, convolution neural networks are one of the best methods for feature extraction. The first step in this method is feature extraction. We carried out this using a convolution neural network. The features that are extracted by this method are mostly related to the edges, colors, and textures of the images. We use the model defined in the second method to extract the features at this stage. The best features that can be selected for this are features that are extracted from the fully connected layer. Our defined model for convolution neural network extracts three features for each image. Then these features are sent as inputs to the next step of the algorithm

#### Classification

At this stage, the features extracted by CNN are classified by different classifiers. Considering that CNN in its late layer uses back propagation algorithms, its speed and accuracy are low. Therefore, by putting this layer in place with a better learning algorithm such as SVM, the speed, and accuracy of this algorithm can be increased. In this paper, we will use different algorithms to examine classification accuracy.

## Experiments and Validation

The accuracy of our model was assessed by evaluating it to differentiate the complex and not very typical phenotypes of the fruiting bodies.

All the experiments were run by 10-fold cross-validation strategy. One round of cross-validation involves partitioning the dataset into complementary subsets, performing the analysis on one subset (i.e. training set), and validating the analysis on the other subset (i.e. validation set or testing set). To reduce variability, 10 rounds of cross-validation were performed using different partitions, and the validation results were averaged over the rounds^33^. The used performance measure is classification accuracy, which is obtained by Eq. (18):

18 $$Accuracy=\frac{{\sum }_{i=1}^{C}\,T{D}_{i}}{{\sum }_{i=1}^{C}\,{T}_{i}}$$

where C is the number of classes which in our case is 22. *TD*_*i*_ denotes true detection of instances in class i and *T*_*i*_ is the total number of instances in class *i*.

The accuracy of our model in the detection of distinct classes is reported by TP and FP values that indicate True Positive Rate and False Positive Rate, respectively.

TP denotes the number of instances, which belong to a class and recognized truly by the proposed modeling as the members of that class. FP denotes the number of instances, which are wrongly recognized as the members of a genus, but they truly belong to other genera. The classification models are trained by the training set and evaluated by the test set which has not been encountered during modeling. This evaluation strategy confirms the ability of the models to predict unseen samples.

Here, we describe an automated image analysis tool that facilitates the identification of Myxobacterial genera independent of need for microscopic or nucleotide sequencing In addition, this can increase the efficiency of the isolation process by optimization of the isolation condition from early on towards retrieving more of the diverse genera instead of compiling the strains that are member of a few limited genera.

Preparing a dataset that includes images with different size and variation in the shape of the fruiting body, results robustness of the proposed method toward shape variations and size of the fruiting bodies due to their maturity, using various culture media or different fields of view.

### Configuration of CNN on myxobacterial pictures-MY22

In Table 3, three different configurations of baseline CNN executed on 22 classes were compared. As one can see, the third configuration provides the best accuracy. Thus, this configuration is used for all of the experiments in our study. However, the best accuracy of CNN is 77.24%.

**Table 3:** Configuration of CNN on Myxobacterial pictures in MY22.

Structure	Feature Extraction Layers	Fully Connected	Accuracy
1	Convolution Layer (fi (2,16), Padding (3,3))	F (O(22))	65.46%
	Max-Pooling (Pol (2,2), Stir(2,2))		
	Convolution Layer (fi (2,32), Padding (3,3))		
	Max-Pooling (Pol (2,2), Stir(2,2))		
	Convolution Layer (fi (2,64), Padding (3,3))		
	Max-Pooling (Pol (2,2), Stir(2,2))		
	Convolution Layer (fi (2,128), Padding (3,3))		
2	Convolution Layer (fi (3,16), Padding (1,1))	F (O(22))	74.246%
	Max-Pooling (Pol (2,2)), Stir(2,2))		
	Convolution Layer (fi (3,32), Padding (1,1))		
	Max-Pooling (Pol (2,2), Stir(2,2))		
	Convolution Layer (fi (3,64), Padding (1,1))		
3	Convolution Layer (fi (3,16), Padding (1,1))	F (O(22))	77.24%
	Max-Pooling (Pol (2,2)), Stir(2,2))		
	Convolution Layer (fi (3,32), Padding (1,1))		
	Max-Pooling (Pol (2,2), Stir(2,2))		
	Convolution Layer (fi (3,64), Padding (1,1))		
	Max-Pooling (Pol (2,2), Stir(2,2))		
	Convolution Layer (fi (3,128), Padding (1,1))		

fi: Filter.

M: Max-Pooling Layer.

Pol: Poll-size.

Stir: Stride.

O: Output.

### Results on myxobacterial pictures- MY25

**Figure 4:**
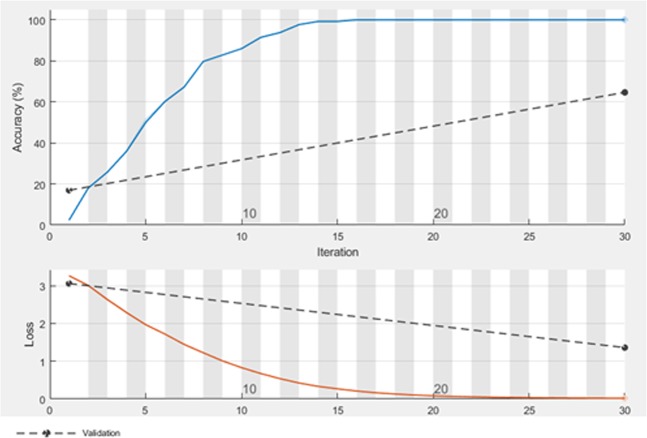
Accuracy and loss of training and validation set of MY25.

The results of MY25 dataset are shown in Table 4. The size of the extracted feature vector for this dataset is 1 × 25. As it can be seen, in this case CNN-MLP has the best result among other algorithms. The accuracy and loss of training and validation set of MY25 when the number of iterations is increased is presented in Fig. 4.

**Table 4:** Configuration of CNN on Myxobacterial pictures in MY25.

Feature extraction	Classifier	Accuracy (%)	Precision (%)	Recall (%)	Parameters
CNN	**MLP**	**80.7**	**100**	**100**	**Learning Rate: 0.3 Hidden Layer: 1 No. of Nodes: 3**
	RBF	25.4	46.2	100	No. of Batches: 100
	SVM	76.84	100	100	Kernel Function: Linear
	XGBoost	74.91	100	100	
	ELM	73.85	75.6	100	Activation Function: Sigmoid No. of Nodes: 100
	CELM	21.54	35.6	100	Activation Function: Sigmoid No. of Nodes: 20
	OSELM	70.77	75.3	78.4	No. of Nodes: 50 No. of train samples: 50 No. of Blocks: 20
	KELM	76.92	100	100	Kernel Function: RBF No. of Nodes: 500
CNN baseline		64.62	100	100	Epochs: 30 Learning rate: 0.01 Iteration per epoch: 1

**Figure 5:**
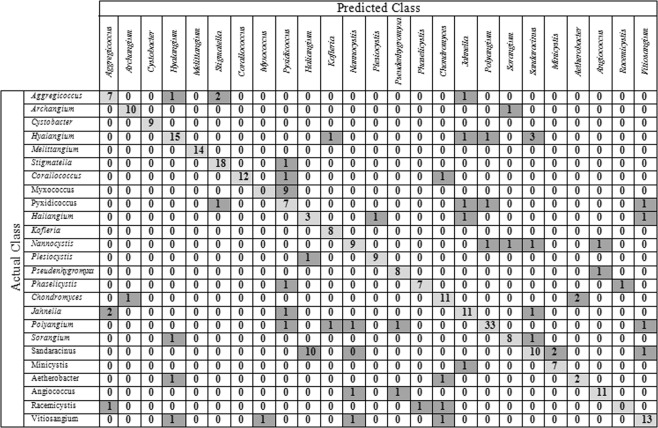
Confusion matrix for CNN-SVM on MY25.

Figure 5 shows the confusion matrix for CNN-SVM on MY25 dataset. Light gray cells show the number of correct classified samples and the dark gray cells related to the number of misclassified samples. As can be seen in the figure, the number of correct classified samples from *Myxococcus* genus is zero and all the samples in this class were classified as *Pyxidicoccus* wrongly. The samples from *Myxococcus* genus include single bodies but the samples from *Pyxidicoccus* genus are colonies. The structure of their fruiting body is similar to each other as both belong to the same family. Although it seems that, the CNN features should be discriminative enough to separate these two classes but in practice, it is not successful. Finding other structures for CNN may reduce the misclassification error.

### Results on myxobacterial pictures-MY22

**Figure 6:**
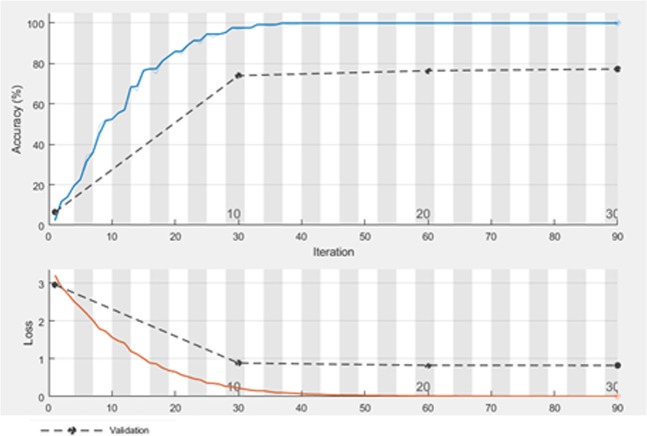
Accuracy and loss on training and validation set of MY22.

The results of MY22 dataset are listed in Table 5. The size of the extracted feature vector for this dataset is 1 × 22. As can been seen, CNN-SVM has the optimum result among other algorithms. The accuracy and loss of training and validation set of MY22 when the number of iterations is increased is shown in Fig. 6.

**Table 5:** Configuration of CNN on Myxobacterial pictures in MY22 dataset.

Feature extraction	Classifier	Accuracy (%)	Recall (%)	Precision (%)	Parameters
CNN	MLP	89.72	96.7	1	Learning Rate: 0.3 Hidden Layer: 1 No. of Nodes: 3
	RBF	31.81	97.3	50.7	No. of Batches: 100
	**SVM**	**89.88**	**1**	**1**	**Kernel Function: Linear**
	XGBoost	86.94	97.3	1	
	ELM	77.24	80.4	79.6	Activation Function: Sigmoid No. of Nodes: 100
	CELM	9.7	24.6	1	Activation Function: Sigmoid No. of Nodes: 20
	OSELM	78.86	82.2	79.8	No. of Nodes: 180 No. of train samples: 300 No. of Blocks: 10
	KELM	85.37	87.3	1	Kernel Function: RBF No. of Nodes: 20
CNN baseline		77.24	1	1	Epochs: 30 Learning rate: 0.01 Iteration per epoch: 3

### Results on myxobacterial MYCategories

**Figure 7:**
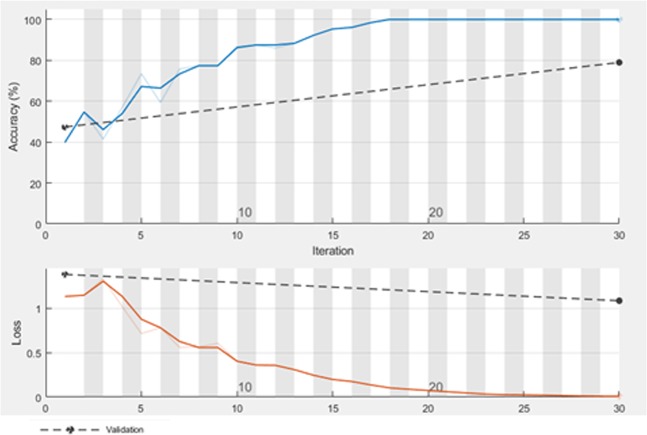
Accuracy and loss on training and validation set of MYCategories.

The results of MYCategories dataset are listed in Table 6. The size of the extracted feature vector for this dataset is 1 × 3. As you see in Table 6 where CNN-SVM has the distinguished result among other algorithms. The accuracy and loss of training and validation set of MYCategories when the number of iterations is increased is illustrated in Fig. 7.

**Table 6:** Performance of different methods on MYCategories.

Feature extraction	Classifier	Accuracy (%)	Precision (%)	Recall (%)	Parameters
CNN	MLP	88.23	92.9	89.6	Learning Rate: 0.3 Hidden Layer: 1 No. of Nodes: 3
	RBF	88.58	92.3	88.6	No. of Batches: 100
	**SVM**	**88.92**	**91.7**	**92.4**	**Kernel Function: Linear**
	XGBoost	86.15	90.8	89.4	
	ELM	77.19	88.4	82	Activation Function: Sigmoid No. of Nodes: 10
	CELM	61.4	70.4	65.3	Activation Function: Sigmoid No. of Nodes: 20
	OSELM	80.7	75.2	82.3	No. of Nodes: 180 No. of train samples: 10 No. of Blocks: 20
	KELM	78.95	79.8	80.4	Kernel Function: RBF No. of Nodes: 10
CNN base line		78.95	86.9	86.9	Epochs: 30 Learning rate: 0.01 Iteration per epoch: 1

**Figure 8:**
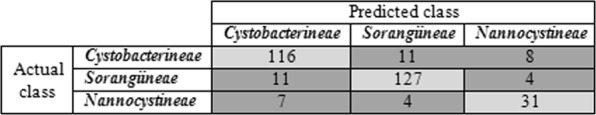
Confusion matrix for CNN-SVM on MYCategories.

Figure 8 shows the confusion matrix for CNN-SVM on MYCategories. Row 1 shows that from 135 samples belong to *Cystobacterineae* suborder. The number of 116 samples were been recognized correctly, 11 samples were wrongly recognized in the suborder *Sorangiineae* and eight samples were displaced as *Nannocystineae* suborder. The results of the Fig. 8 show that the probability of wrong recognition between *Cystobacterineae* and *Sorangiineae* is higher compared to others.

## Conclusion

Application of pattern analysis in microbiology and biotechnology is accelerating the speed and accuracy of the procedures and practices. By increasing the number of Myxobacterial genera especially with similar fruiting bodies, the challenge in their instant recognition has emerged recently. The conventional techniques that involve observation of the macromorphology and its characteristics such as color, shape, and pigment is considered acceptable criteria for bacterial identification in some groups of bacteria. However, morphology oriented methods have some impediment, for instance, they usually need proficient personnel who have a solidified knowledge on the morphology and taxonomy of Myxobacteria.

In recent decades, automated methods have been applied in laboratories for rapid identification of bacteria. Using machine learning methods, a combination of experience and technology in the task of bacterial identification will be provided. It is anticipated that in the near future the number of new genera in all microbial taxa will dramatically be increased. This fact has been observed in the last five years with the introduction of six new genera that do not have the peculiar characteristic shape of fruiting bodies that are even named as pseudo fruiting bodies. However identification of some myxobacterial genera such as *Stigmatella, Chondromyces, Corallococcus, Myxococcus, Cystobacter*, and *Archangium* is rather easy by observation, there are challenges in case of some genera that produce a amorphousand disorded forms of the fruiting body like *Kofleria, Jahnella*, *Enhygromyxa*, *Plesiocystis*, *Pseudenhygromyxa* and *Haliangium*, etc.

Automated imaging and analysis have the potential to improve the duration and accuracy of identification of Myxobacteria required for a variety of ecological and biotechnological projects.

The introduced automated recognition can enable an analysis of individual fruiting bodies, taken over time or all presented at a single image. Additionally, the classification of distinct fruiting body shapes based on image-derived features was independent of whether pictures are colored or on a grey scale. Phenotypic changes in the morphology of fruiting body due to being in variant maturity stage can be expressed as minor changes in feature space, which can be corrected and attributed to the respected suborder.

In this study, a platform that uses stereomicroscopic image analysis and pattern recognition to differentiate between 30 genera of Myxobacteria was developed based on the phenotypic signatures. The images of the newly discovered genera of Myxobacteria can be added to the dataset and the proposed structure can be retrained to discriminate between genera automatically.

In this work, the images were gathered from different resources, captured by various cameras with disparate resolution and lightening environment. Image capturing with the similar situation can enhance the accuracy of the recognition result and hence the efficiency of automated identificatio system.

## Data Availability

The analysis data of this study are available from the corresponding authors for follow up studies.
